# Unleashing innovative cross-organ fibrosis therapies by harnessing the omics revolution 

**DOI:** 10.1172/jci.insight.200155

**Published:** 2026-07-08

**Authors:** Cynthia Lebeaupin, Katelyn L. Donahue, Ken Dower, Thomas A. Wynn, Kevin M. Hart, Thomas Fabre

**Affiliations:** Pfizer Inc., Cambridge, Massachusetts, USA.

## Abstract

Fibrosis is a major cause of mortality and morbidity worldwide with limited therapeutic options. Our understanding of fibrosis has significantly improved and led to the identification of “core” fibrogenic mechanisms that fuel a self-sustaining vicious cycle following the initial insult. The fibrotic niche is the result of complex cellular and molecular interactions that need to be disrupted to achieve transformational therapies. In this Review, we describe the current understanding of fibrogenic mechanisms, the progress and limitations of omics approaches in the identification of novel fibrotic pathways, and advances in therapeutic modalities that all together have the potential to unleash innovative cross-organ antifibrotic therapies.

## Introduction

Progressive tissue fibrosis is a hallmark of many chronic human diseases. Despite fibrosis being a major contributor to morbidity and mortality, highly effective antifibrotic therapies are lacking. The reasons underlying this paucity include an incomplete understanding of pathogenic versus protective mechanisms, challenges in identifying tractable therapeutic targets that have acceptable safety margins, and a focus on addressing factors that initiate disease and upstream inducers of epithelial cell damage rather than understanding the core mechanisms that cause normal tissue remodeling following injury to transition to pathological scar formation.

Scar tissue, which characterizes all progressive fibrotic diseases, triggers additional pathogenic changes in the tissue that over time affect nutrient and waste diffusion (e.g., gas exchange, bile); destroy blood vessels, leading to more severe hypertension (e.g., coronary artery disease, esophageal varices, pulmonary hypertension); and impair normal cell function. These effects together disrupt normal tissue architecture, leading to progressive organ stiffness and dysfunction, as seen in chronic kidney disease, interstitial lung disease (ILD), idiopathic pulmonary fibrosis (IPF), myocardial fibrosis, and liver cirrhosis (1, 2). These tissue changes progressively worsen disease outcomes by exacerbating cell death in an already-stressed cellular environment, driving additional inflammation and promoting further expansion of scarring in a self-sustaining feedback loop. These processes appear to be augmented with age-associated disease in liver (3, 4), lung (5), kidney (6), and heart (7), with aging causing changes in fibroblasts that decrease their support of homeostatic niches, increase activation after injury, and reduce contraction during resolution (8). Indeed, fibrosis is a clear independent correlate for patient outcomes and survival in cirrhosis and many other chronic inflammatory diseases (9, 10).

However, the belief that extensive scarring in diseased tissue is progressive and often irreversible has been challenged by the advent of curative hepatitis C agents, which have convincingly demonstrated regression of fibrosis in some patients, despite confirmatory follow-up studies largely relying on noninvasive measurements (11). Similarly, data from patients undergoing gastric bypass (12) or those in trials of metabolic dysfunction–associated steatohepatitis (MASH) therapies (13) suggest that even highly efficacious mechanisms of reducing epithelial death and disease activity score (NASH activity score [NAS]/MASH activity score) may be insufficient to induce robust fibrosis resolution in a large proportion of patients. Multiple treatments have shown improved fibrosis in only a small percentage of patients — resmetirom: ~12% placebo adjusted (14), semaglutide: 14.4% placebo adjusted (15), obeticholic acid: 11% placebo adjusted at high dose (16), pegozafermin (modified FGF21): 20% at high dose (17), efruxifermin: 10% and 16% placebo adjusted at top two doses in cirrhosis and 21% placebo adjusted in patients with F2/3 MASH (18, 19). Indeed, complete reversal of disease likely occurs in only a small fraction of treated patients, with those with more severe fibrosis prior to treatment showing little to no reversal and many displaying an ongoing worsening of disease (20). Despite treatment of the underlying cause, many patients with fibrosis remain at increased risk for mortality and tumor development (reviewed in refs. 21–24). Collectively, it is increasingly clear that patients will require bona fide antifibrotic therapies as standalone or in combination with epithelium-centric treatments to achieve robust resolution of their pathological fibrosis. Reversing the function of or eliminating senescent cells and other pathogenic cell players that respond to tissue damage and identifying critical central mechanisms that induce progressive fibrogenesis are enabled by advances in biotechnologies, which, if leveraged correctly, may drive a wave of innovative target identification and transformative pan-organ therapies.

## Mechanisms of fibrosis

Our current understanding of fibrosis biology stems from foundational mechanistic discoveries and experimental frameworks, which remain the bedrock of omics-driven discovery. Omics technologies expand our ability to map molecular landscapes, while serving to reinforce and contextualize discoveries first made through classical hypothesis-driven studies. Many of these concepts are introduced and synthesized in this Review as the launching point for cross-organ therapeutic innovation.

### Insult to injury.

**Figure 1:**
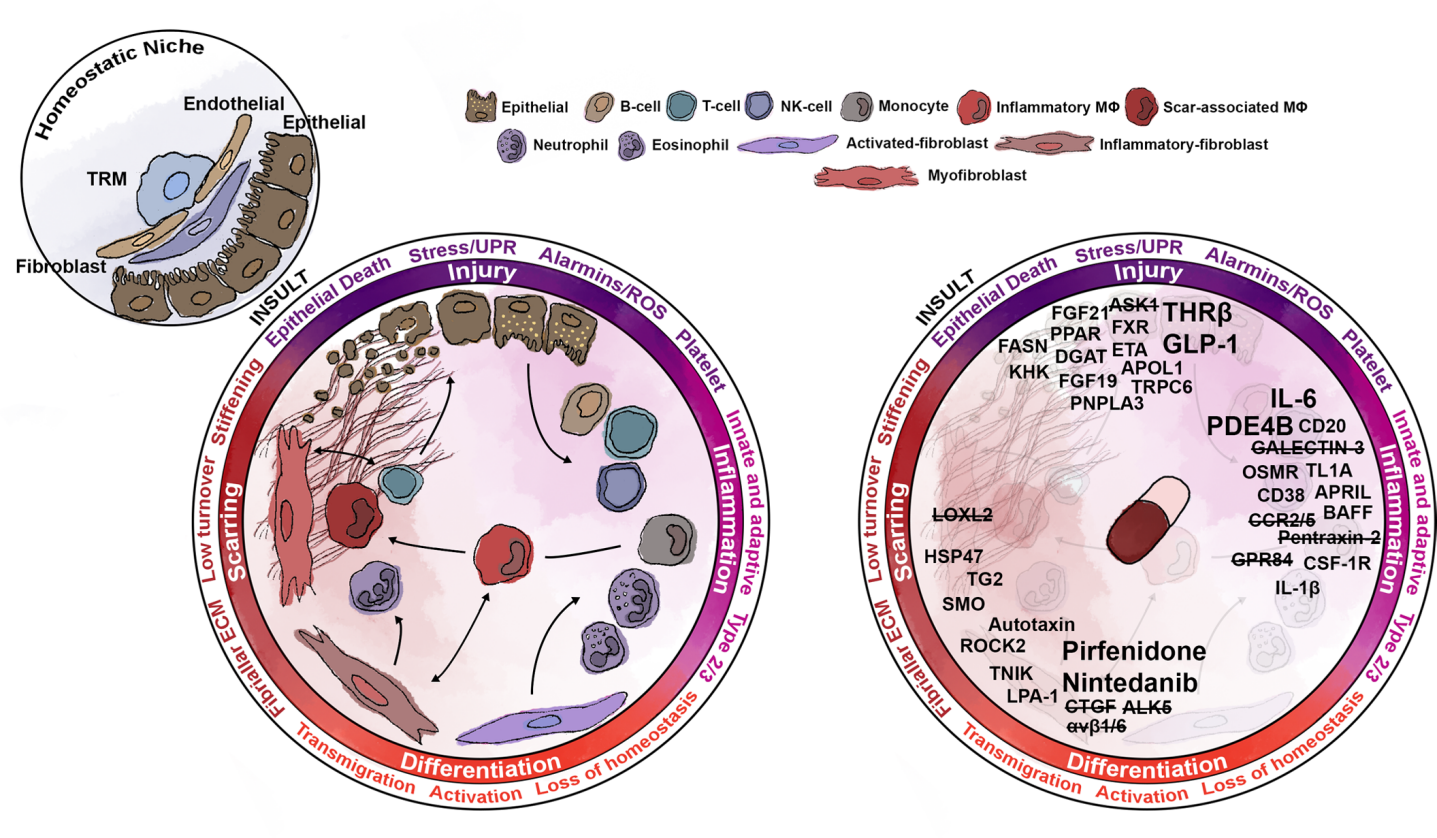
The feed-forward cycle of injury, inflammation, differentiation, and scarring in chronic fibrotic disease. Chronic fibrotic diseases across organs are driven by a conserved, self-perpetuating cycle of injury, inflammation, cell differentiation, and scarring. An insult (e.g., environmental toxins, pathogens, genetic predisposition) begins the cycle. Insult leads to injury via cellular stress that activates the endoplasmic reticulum unfolded protein response (UPR), releases alarmins and mitochondrial reactive oxygen species, and culminates in epithelial cell death. These events initiate the inflammatory phase due to platelet infiltration, recruitment of innate immune cells (e.g., monocytes/macrophages, neutrophils, dendritic cells, DCs), and activation of adaptive immune responses (Th2 cells, Th17 cells, Tregs, B cells), orchestrated primarily by a milieu of type 2 and type 3 cytokines. This immune activation induces the loss of tissue homeostasis and primes the fibrotic niche. During the differentiation phase, endothelial cells undergo defenestration with loss of endothelium integrity and a switch to a pro-inflammatory/recruitment phenotype, while resident macrophages are depleted and replaced by monocyte-derived profibrotic/pathogenic macrophages. Fibroblasts differentiate into myofibroblasts, acquiring contractile and extracellular matrix–producing (ECM-producing) phenotypes aimed first at injury resolution and tissue repair. If resolution and repair are unsuccessful, the scarring phase is perpetuated by excessive ECM deposition, reduced matrix degradation due to MMP/tissue inhibitor of metalloproteinases (TIMP) imbalance, and increased tissue stiffness that contribute to scar blockade. These changes impair epithelial regeneration, restrict perfusion, and reinforce the fibrotic microenvironment, thereby alimenting the cycle. Failure to interrupt this cycle leads to progressive fibrosis and organ dysfunction. The right panel shows currently approved antifibrotic therapies (in bold and larger font: Esbriet [pirfedinone] for IPF, Ofev [nintedanib] and Jascayd [nerandomilast] for IPF/progressive pulmonary fibrosis, Actemra [tocilizumab] for systemic sclerosis–ILD, Rezdiffra [resmetirom] and Wegovy [semaglutide] for MASH; in strikethrough: active or stopped phase II/III assets and their relative impact on the “core” fibrotic mechanisms of injury, inflammation, differentiation, and scarring).

Fibrosis is a maladaptive wound-healing response driven by a self-sustaining, four-stage cycle (Figure 1). Fibrosis often begins with epithelial (or, in some cases, endothelial) injury triggered by a variety of insults, including environmental toxins, pathogens, immune reactions, lifestyle factors, or genetic predispositions (as reviewed in refs. 25–27). Genomic studies in individuals with organ-specific fibrosis have identified susceptibility loci (28), such as *PNPLA3* and *HSD17B13* in liver (29, 30), *TERT* (31), *MUC5B* (32), in lung, and *SLC2A12* and *HFE* in the heart (33). The resulting injury triggers cell stress and often death of vulnerable cell types, such as hepatocytes in the liver or proximal tubular epithelial cells in the kidney, due to metabolic or hypoxic insults. Early injury across organs involves epithelial disruption, microvascular damage, and in some cases microbiome imbalances that together trigger inflammation (34, 35).

Cellular endoplasmic reticulum stress (36) activates the UPR and integrated stress response (37), whereas mitochondrial dysfunction leads to ROS production (38). Both pathways can initiate NLRP3 inflammasome activation (39, 40) and drive sterile inflammation, releasing damage- or pathogen-associated molecular patterns and alarmins, such as IL-33 (41) and HMGB1 (42, 43). Cell death pathways (as defined in ref. 44), namely apoptosis and pyroptosis, engage in crosstalk with innate immunity and compromise epithelial integrity (45). These conserved features across different diseases are actively being investigated as therapeutic targets: e.g., pan-caspase inhibitor emricasan (46), necroptosis-related RIPK3 (47), and pyroptosis-related gasdermin D (48). As the nature of the initiating insult or predisposition may remain organ specific, and sometimes unknown (idiopathic), omics approaches may connect disparate biological threads into coherent, targetable pathways across organ systems (49).

### The immune cascade from inflammation to scarring.

Following injury, inflammation is rapidly initiated by platelet degranulation, consisting of the release of bioactive molecules, including growth factors (e.g., TGF-β, PDGF), adhesion molecules, cytokines/chemokines, and other mediators that prime endothelial cells and fibroblasts, enhance vascular permeability, and promote leukocyte recruitment (50). Concomitantly, activated platelets can upregulate surface CD62P (P-selectin) and CD40L (CD154), release α-granule cargo, and expose phosphatidylserine, collectively creating a procoagulant scaffold that amplifies tissue factor-driven thrombin generation. A platelet-dependent activation of the coagulation cascade promotes fibrin deposition and stabilizes the provisional wound matrix, while facilitating platelet-leukocyte interactions that shape early inflammatory and stromal responses (51, 52). Activated platelets (51) and fibroblasts secrete cytokines and chemokines that attract neutrophils and monocytes, which polarize and differentiate into pro-inflammatory macrophages. Crosstalk between macrophages and stromal cells is a core mechanism in fibrogenesis. An inflammatory niche is established, particularly skewing toward type 2 (53, 54) and type 3 inflammation (55, 56), also reviewed (57). Type 2 inflammation, characterized by IL-4, IL-5, and IL-13 cytokines, promotes fibroblast activation and macrophage polarization (58). In parallel, type 3 inflammation, characterized by IL-17 and IL-22/IL-23, enhances fibroblast TGF-β1 receptor expression and neutrophil recruitment (55, 59, 60). Neutrophils defend against insults through phagocytosis, degranulation, and the formation of neutrophil extracellular traps, which are linked to organ fibrosis (61–63). Adaptive immune cells are also recruited, though their role as central mediators (as opposed to bystanders) across fibrotic indications is less clear, as the antigens that drive their activation are not well characterized. Calculating the neutrophil-to-lymphocyte ratio was presented as an accessible marker of immune imbalance, linked to disease progression, hospitalization risk, and mortality in pulmonary fibrosis (64). Nonetheless, lymphocytes can reinforce certain inflammatory niches (65), as through the autoaggressive T cell phenotype (66). Autoantibodies from B cells — long seen as diagnostic markers — contribute to chronic damage in kidney nephropathies, rheumatoid arthritis, and systemic sclerosis (67), which can lead to interstitial fibrosis. In contrast, Tregs and NKT cells tend to suppress fibrogenic pathways, but their impact as either protective or pathogenic remains complex and context dependent (68–70). Rebound inflammation can occur in response to some antifibrotic treatments and may further exacerbate the mechanisms that promote fibrosis. Thus, a deeper understanding of the cellular mediators and pathways that promote, terminate, and reverse fibrosis is needed to develop the most effective antifibrotic treatments (53, 71–73).

The chronic presence of pro-inflammatory cytokines, such as IL-1β, IL-6, and TNF-α, sustains inflammation and primes fibroblasts for differentiation (74). Fibroblasts lose their quiescent, homeostatic identity and acquire a contractile, ECM-producing phenotype as myofibroblasts (25) in a transition further orchestrated by TGF-β1 (75), IL-11 (76), and PDGF (77). Moreover, defects in macrophage efferocytosis can perpetuate disease, as homeostatic clearance of apoptotic cells is critical in inflammation and fibrosis resolution (78–83). With sustained fibrosis, myofibroblasts express α–smooth muscle actin (α-SMA) and secrete large amounts of collagen and fibronectin, contributing to scar formation that is reinforced by mechanical cues (84). Excessive ECM deposition, primarily collagens I and III, disrupts normal tissue architecture and function. This accumulation is exacerbated by underlysis or reduced ECM degradation due to an imbalance between MMPs and their inhibitors (TIMPs) (85–87). The resulting ECM stiffness not only prolongs fibroblast activation, as driven by YAP/TAZ (88–90) with PIEZO activation (91) or α_v_β integrins (92) and NOTCH (93, 94), but also impairs epithelial regeneration (95, 96), immune cell mobility, and perfusion/nutrient delivery via defects in glucose, lipid, or amino acid metabolism (97). More than a consequence of injury, fibrosis nurtures a dynamic immunological and cellular bottleneck that traps tissues in a cycle of chronic dysfunction and impaired repair.

### Myeloid/stromal dynamics in the fibrotic niche.

Shared and divergent features between fibrosis across different organ systems, as reviewed (25), reveal a common interplay between fibroblasts and macrophages in both tissue homeostasis and fibrosis (98). Tissue-resident macrophages, which can arise from yolk sac or bone marrow, typically self-renew and contribute to tissue homeostasis (99–104). In response to injury, the resulting inflammation and fibrosis often lead to a depletion of resident macrophages (105), which are replaced by monocyte-derived macrophages that either rescue tissue sentinel function (106) or perpetuate inflammation and fibrosis (107, 108). Infiltrating monocytes differentiate into a pathogenic population of SPP1^+^ macrophages (109–112), also reported as scar-associated macrophages and lipid-associated macrophages, that appear to be largely conserved across fibrotic diseases. Indeed, an integrated human single-cell RNA-seq (scRNA-seq) atlas curated from liver and lung fibrosis patient data identified a subset of macrophages coexpressing five markers (SPP1, GPNMB, FABP5, CD63, and CD9) that is enriched at the edges of fibrotic scars and closely associated with activated mesenchymal cells and neutrophils producing IL-17A, GM-CSF, and MMP9 (109). The monocyte to pathogenic macrophage transition during liver disease was recently linked to NOTCH/RBPJ signaling (113). It is increasingly clear that macrophages are heterogeneous and require more elaborate nomenclature than previously used (114). Macrophage identity is, and continues to be, shaped by developmental origin, tissue-specific cues, and environmental context (115). Understanding the spatiotemporal dynamics of monocyte-to-macrophage transitions after injury is critical for designing strategies to target pathogenic macrophages in fibrosis.

Aside from the well-established role of α-SMA^+^ myofibroblasts in pathological ECM deposition, a human fibroblast atlas revealed surprisingly diverse subtypes of fibroblasts where distinct pathological conditions reshaped fibroblast transcriptomes, leading to the description of myofibroblast subsets, like LRRC15^+^ or MMP1^+^ fibroblasts, that form potent immunosuppressive niches (116). Certain resident fibroblasts are important sentinel cells in all tissues that produce leukocyte-recruiting chemokines following injury or infection (117). Similar to macrophages, fibroblasts are dynamic in their identity, spatial distribution, and function, as explored by transcriptomic characterization of lineage tracing models and pseudotime analyses in various fibrotic organs (118). Spatial transcriptomics identified injury-specific microenvironments defined by localized epithelial-fibroblast-immune interactions, such as *Clcf1/Crlf1* (IL-6 family member) (116) or *Jag1/Notch3* (119) signaling and *Snai1* (120) in the kidney, that orchestrate inflammation and fibrosis following acute injury. Fibroblasts may become locked in a profibrotic state because of epigenetic reprogramming of epithelial-mesenchymal transition, driven by regulators that inhibit *CDH1* (encoding key adherens junction protein E-cadherin) or enhance *BRD4*-associated (121) and *MEOX1*-associated (122) pathways, making reversal challenging (123). Fibrosis is simplified as a linear progression of injury, inflammation, and fibrotic remodeling. In reality, these occur cyclically and asynchronously across spatially distinct niches within the organ.

## The evolving landscape of omics studies in fibrosis

The fibrosis research community is rapidly adopting high-resolution and high-parameter omics technologies in applications, such as scRNA-seq, single-nuclei RNA sequencing (snRNA-seq), spatial transcriptomics, Epi Multiome assay for transposase-accessible chromatin with sequencing (ATAC-seq), ChIP-seq, and more, resulting in robust, publicly available datasets that are revealing novel targets and core profibrotic pathways, with innovative artificial intelligence (AI)/machine learning (ML) technologies likely to rapidly advance our understanding and treatment paradigms for fibrotic disease in the very near future. The resulting effects and current pitfalls of omics integration to fibrosis studies need to be evaluated. The implementation of omics technologies in fibrosis research has evolved with new technical and computational methodologies (as reviewed in ref. 124).

### Wrangling cells of interest.

The study of fibrosis with single-cell transcriptomics continues to be optimized because of challenges in capturing intact-cell scRNA-seq for some tricky populations (e.g., hepatic mesenchymal cells). To circumvent this issue, investigators have implemented snRNA-seq to yield datasets with increased stromal coverage (125). In human livers, tumors, or fibrotic kidneys, snRNA-seq showed increased recovery of several cell types, including mesenchymal cells, mesangial cells, podocytes, and endothelial cells, that were poorly detected by scRNA-seq (126–128). Although snRNA-seq data displayed reduced technical noise compared with scRNA-seq data, snRNA-seq can be combined with gradient centrifugation and cell- or nucleus-based sorting to optimize representation of mesenchymal cells and other challenging populations (129). Furthermore, snRNA-seq is compatible with fixed and frozen samples, thus enabling greater flexibility with tissue banking approaches. While snRNA-seq improves sensitivity of epithelial and mesenchymal cell coverage, scRNA-seq has superior profiling of immune cells, making a combination of both technologies ideal to compare the healthy and diseased tissue landscape, as reviewed (125).

### Mapping fibrosis with transcriptomic atlases.

Transcriptomic atlases, which combine multiple datasets (109, 118, 130–133), have the fundamental benefit of including a great number of cells, organs, and patients, therefore capturing rare and heterogeneous cell populations. Increased power from transcriptomic atlases and trajectory inference more readily identifies relationships between phenotypes and transitional populations.

However, transcriptomic profiling is complicated by varying patient sample source and procurement, sample preparation (e.g., tissue digestion and batching), sequencing technologies, sequencing depth, and genome alignment. This hinders uniform transcriptomic profiling due to missing data, collinearity, and dimensionality variabilities (49, 134). Bearing in mind these covariates, multiple approaches have been taken to perform accurate dataset integration (132, 134, 135). The resulting atlases highlight similarities and differences in the annotation of key pathology-associated cell subsets (such as SPP1^+^ macrophages) (109, 135) or dive deeper into broadly annotated cell types (e.g., DCs divided into DC1/DC2) (136). Combining multiple fibrotic compartments (liver, lung, heart, kidney) has revealed potential interventions for cross-organ therapies (109, 137).

### Multiomic approaches unlock understanding.

Although transcriptomics offers a wealth of information, it has limitations, such as cell population recovery, low-expressed gene detection, and innate differences in RNA/protein abundance (138). Many advanced applications extract predictions without conclusively demonstrating cellular activity from transcriptomic data, namely pathway activation, transcription factor activity (139, 140), and cell-to-cell communication (141). Better predictions can be made by combining multiple omics modalities. For example, cellular indexing of transcriptomes and epitopes with sequencing (CITE-seq) combines surface protein abundance measurements via antibody binding with gene expression to profile both transcriptome and proteome (137, 139, 142). Furthermore, populations of interest can be identified by unbiased RNA-seq and localized with spatial transcriptomics of disease area versus healthy parenchyma (143). Combining sc/snRNA-seq data with spatial transcriptomics to maintain the depth and breadth of transcriptomic profiling with the distribution of ligands and receptors improves cellular communication predictions (144, 145). Multiome/scATAC-seq are other powerful techniques that complement transcriptomic data (102, 137, 139). These technologies detect transcription factor activity and inform on cell ontogeny by profiling the epigenome (146, 147), which is beneficial to translate conclusions from mouse lineage tracing studies to human samples. The combination of epigenome and transcriptome informs on a cell’s past experiences and origin as well as its present role in disease as snapshots into dynamic disease (102). Realistically, longitudinal patient samples are necessary but challenging to obtain, particularly from patients with fibrosis compared with those with cancer, due to the invasive nature of sample collection needed to accurately characterize disease progression.

### Omics in the driver’s seat of functional biology.

The current application of omics technologies provides observations that are phenotypic rather than functional in nature. Molecular pathway signatures established in vitro are inherently biased by cell type and the lack of complex cell-to-cell interactions, thus requiring contextualization for broader biological relevance. New systems are emerging for omics (e.g., Perturb-seq, ref. 148, and Drug-seq, ref. 149) to accurately characterize the dynamic disease environment. For example, spatial transcriptomic profiling of various CRISPR-edited lung cancer cells identified *Tgfbr2* as a key modulator of tumor growth in mice (150). A combination of in vitro human CRISPR screen with transcriptomic atlas revealed gene perturbation relationships, with *ZEB2* as a master switch controlling tumor-associated macrophages (151). Transcription factor perturbations ex vivo by CRISPR activation revealed *KLF2*, *KLF4*, and *PLAGL1* as key regulators of pro-inflammatory fibroblasts (140). Implementing omics approaches in high-throughput perturbation screens to map out healthy versus diseased states will continue to uncover essential mechanisms of disease, and consequentially, potential therapeutic opportunities.

## Targeting profibrotic mechanisms: past, present, and future

Despite significant progress in our biological understanding of fibrosis, the number of approved antifibrotic therapies can be counted on two hands (Figure 1). Most importantly, none of the approved therapies have demonstrated pan-tissue activity in the clinic, because they target a mechanism that is disease specific, or expansion to other indications may be limited by tolerability considerations. This underscores the challenge for developing antifibrotic medicines with multi-indication potential. Approved therapies largely slow fibrosis progression rather than causing substantial reversal of fibrosis. However, at this current inflection point in multiomics technologies, new modalities, and AI/ML, the tide may slowly shift.

### Elementary steps for antifibrotic therapies.

Drug modalities for the treatment of fibrosis have principally relied on small and large molecules. Antifibrotic therapies are developed around antagonism of the key pathological features of disease (Figure 1) or agonism of proresolution/quiescent pathways. Development includes building confidence in rationale around a predefined therapeutic hypothesis using preclinical models. These models span 2D (primary cells) and 3D (organoids) in vitro, in vivo, and ex vivo models, such as tissue slices (152). Drug development also involves establishing a sufficient therapeutic index (benefit versus risk), clinical biomarkers, and robust clinical trial design. These steps are critical to ensure clinical success in fibrosis trials, which are often long, difficult, and costly. Early intervention in fibrotic disease remains a major challenge, as most current imaging modalities lack the sensitivity to detect early-stage fibrogenesis, and patients are frequently asymptomatic during this window. Consequently, most clinical trials enroll patients with more advanced fibrosis in order to reduce trial duration and maximize the likelihood of detecting a measurable therapeutic delta, particularly given the detection limits of current endpoints and the narrow therapeutic index of many core fibrotic pathways. The development of sensitive, mechanism-informed biomarkers, potentially enabled by multiomic approaches, represents a holy grail for enabling earlier diagnosis, rational patient stratification, and credible evaluation of therapeutic impact.

The currently approved therapies for IPF/ILD, nintedanib, pirfenidone, and nerandomilast (153), are complex kinase inhibitors and PDE4B blockers that target growth factor signaling (nintedanib target PDGF, for example, is considered a core fibrogenic pathway) (154). While these drugs meet all the initial criteria, they fail to demonstrate safe and significant efficacy outside of the lungs and therefore cannot be considered pan-antifibrotic drugs. This limitation stems from an unclear mechanism of action due to the polypharmacology and low tolerability of such molecules (155). In contrast, despite their robust efficacy in preclinical models across tissues, both small (higher polypharmacology) and large molecule (more specific) TGF-β inhibitors have failed in the clinic due to toxicity and lack of tolerability. This led to development of new modalities around TGF-β biology to improve safety while maintaining efficacy, which includes antibodies against active TGF-β1, inhibition of specific TGF-β isoforms (1, 2, or 3), and the various pathways and mechanisms that contribute to TGF-β activation (e.g., α_v_ integrins) (92, 156, 157). Integrin inhibitors carried both significant efficacy and improved tolerability in preclinical models but recently failed in the clinic due to worsening of disease (158). Finally, several mechanisms that were safe and well tolerated failed to meet their clinical endpoints despite showing promising positive trends in trials (e.g., anti–IL-13, CTGF), speaking to the importance of well-designed and statistically powered trials with acceptable endpoints (159–161). Thus, combining safe and well-tolerated mechanisms may be necessary to achieve significant outcome in patients.

### Connecting data with AI/ML.

Recent technological innovations, like gene editing, molecular glues, antibody-conjugates (162), multifunctional antibodies, lipid nanoparticles (LNPs), and AI/ML are enabling and accelerating the development of new therapies, in part due to the transformative impact of the 50-year discovery of mAb technology (163). In the era of multiomics datasets, it is attractive to quickly query data to identify and develop safe and innovative therapeutics. The number of data packages and models developed to mine genetic, transcriptomic, and proteomic datasets for target identification, structure-activity relationships, peptide molecular design, epitope mapping, and protein-protein interactions continues to expand at a breathtaking pace (164–166). In pulmonary fibrosis, using an AI/ML pipeline, a small molecule kinase inhibitor targeting TRAF2 and NCK interacting kinase (TNIK) was identified, preclinically validated, synthetized, and nominated for clinical use within 18 months (167). TNIK was selected based on a so-called druggability filter, which is designed to assess whether a target has therapeutic potential. While clearly advantageous for speed, few targets are considered undruggable in this era of ever evolving therapeutic modalities that include degraders (i.e., PROTAC, LYTAC), molecular glues, proton pump inhibitors, macrocyclic peptides, siRNAs, ADCs, LNPs, modified mRNAs, splicing modulators, and multifunctional antibodies, which together have ushered in a major paradigm shift in the pharmaceutical industry that used to rely heavily on traditional small molecules (168). Indeed, the TNIK-targeting molecule showed early signs of efficacy 12 months later in a phase IIa trial in pulmonary fibrosis, highlighting the potential of these innovative approaches to drug development (167, 169). Time will tell whether targeting TNIK will be transformational for patients, as the real hurdle for antifibrotic drug development has often been the transition from the smaller phase II studies to much larger phase III clinical trials (170, 171). Moreover, a virtual lab of human-created AI research scientists recently developed a novel computational pipeline for drug discovery that designed nanobodies with promising binding profiles to SARS-CoV-2 variants (172). As such, it is important to consider that current AI/ML models are as good or accurate as the data feeding them. The next wave of datasets from perturbation (gene-editing or drug based) screening in vitro and ex vivo from clinical trials will improve the foundation of knowledge needed to build better predictions (148, 149, 173–176). As an example, gene signatures generated from human precision-cut lung slices treated with antifibrotic compounds were compared with an existing comprehensive IPF atlas for clinical relevance (177). A new frontier of perturbation sequencing will come from in vivo screens that better recapitulate the complex interactions between cells in the fibrotic niche. This iterative loop of data from perturbation and drug screening will more efficiently expand fundamental knowledge and therapy development. Ultimately, the successes and failures of such approaches in the clinic will refine models to develop the next generation of therapeutics.

### Adding strength through combination therapies.

**Figure 2:**
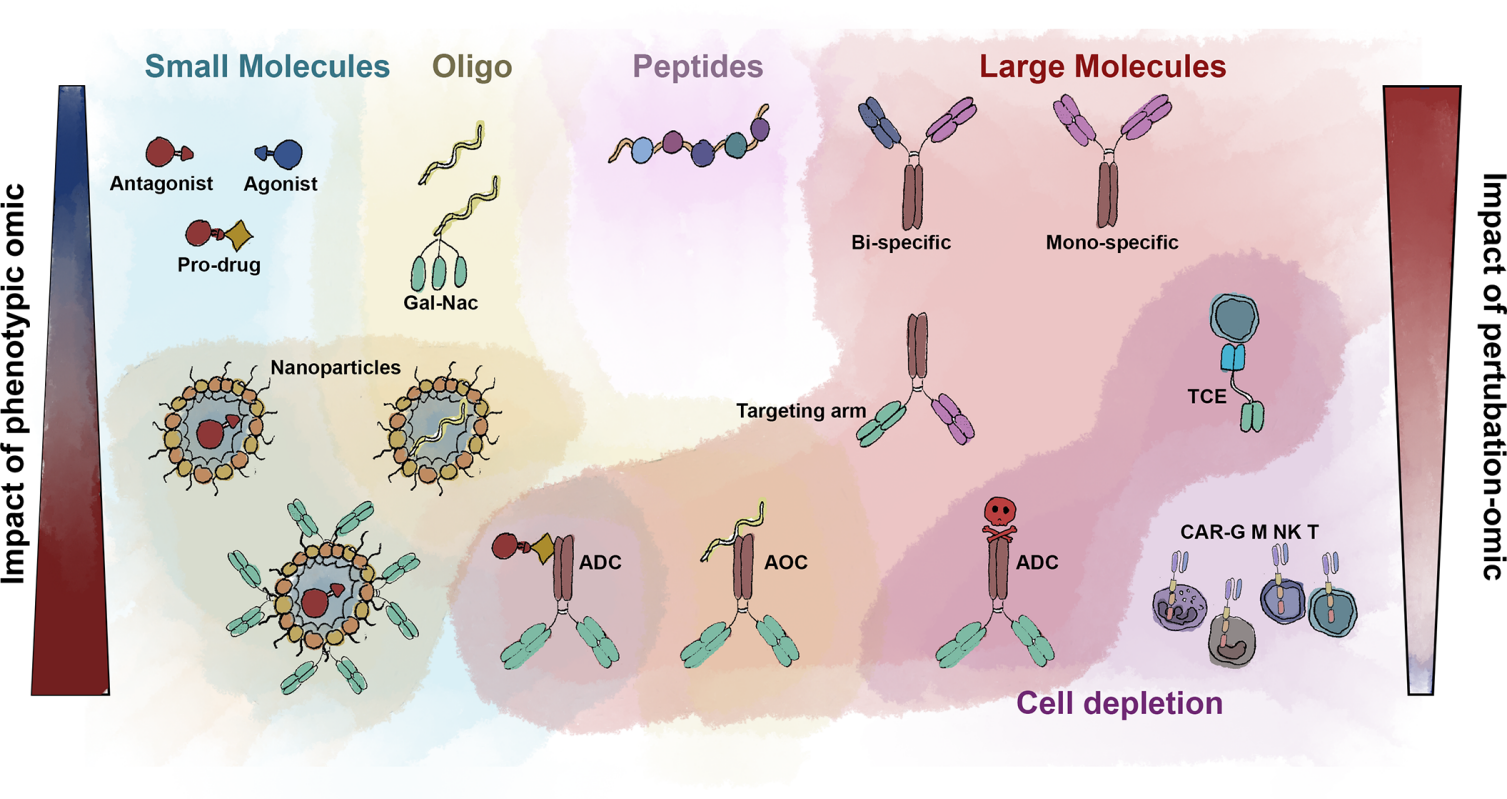
Representative overview of existing and novel therapeutic modalities. Therapeutic modalities are organized from small (left) to large (right) molecules. Untargeted therapeutics are located on the top side of the figure while the bottom part represents cell-specific modalities. Each light color shading represents a specific modality: small molecules (blue), oligos (yellow), peptides (pink), large molecules (red), and cell depletion strategies (purple). Watercolor overlap highlights therapeutics combining different modalities, such as antibody drug-conjugate (ADC) or antibody oligo-conjugate (AOC). Nanoparticles deliver both small molecules and oligos can be further directed to relevant cell types when combined with a targeting arm (green). The left and right gradients show the relative impact (blue low, red high) of phenotypic omics and perturbation screen on the discovery of novel therapeutic targets (top) versus cell-targeting strategies (bottom).

Combination therapies have significantly improved patient outcomes in oncology and are being applied to dermatology, rheumatology, gastroenterology, and respiratory diseases (178–180). In contrast, fibrosis has been slower to evaluate this strategy beyond MASLD/MASH. Metabolic drugs and antisteatotics were combined with antiinflammatory/antifibrotic molecules to treat patients with late-stage disease (181, 182), but no beneficial effects were observed over monotherapies (183). Recently, a combination of an FGF21 analog and GLP-1 agonists demonstrated additional antifibrotic efficacy while maintaining significant improvement of weight loss and NAS (184). Reanalysis of preclinical, clinical, and safety data with AI/ML could help design combination therapies targeting nonoverlapping and orthogonal fibrotic pathways. Progress made on protein engineering (such as bi- or multispecific antibodies) (185) opens a door to therapies affecting multiple pathways while maintaining higher specific and lower off-target effects compared with small molecules (Figure 2). This is a clear trend for biologics across therapeutic areas where transformational efficacy may be achieved with more complex biologics. This approach will not be restricted to large molecules, as high-horsepower small molecules that target core fibrogenic mechanisms can be combined with other molecules that target different disease parameters.

### Creative targeting of master mechanisms.

Central pathways in fibrosis, such as TGF-β, YAP/TAZ, and NOTCH, also participate in homeostasis, normal wound healing, and systemic processes, thereby limiting therapeutic index (186, 187). This limitation may be overcome using tissue- or cell-specific delivery systems. For instance, reformulation of nintedanib into an aerosol is being explored to enhance lung exposure and limit off-target effects (188, 189). A secondary approach is to engineer inactive compounds that require proteolytic activation by proteases expressed specifically in disease areas, known as protease “unmasking” (190). Unmasking is not new; however, significant advances in chemistry have increased its potential beyond oncology to fibrosis because of the similar proteolytic nature of the fibrotic niche. Cell-targeted strategies (Figure 2) are expanding to selectively deliver therapeutic payloads (e.g., small molecules, oligonucleotides: siRNA, mRNA) with nanoparticles or antibodies. For nanoparticles, cell specificity can be achieved by manipulating sugar and lipid content or adding targeted antibodies against a cell type of interest (191). Antibody drug- or oligo-conjugates (ADCs and AOCs, respectively) are gaining traction as viable modalities to treat chronic diseases but require proper identification of cell surface targets that are specific and have the right molecular properties (192, 193). Despite these challenges, several interesting approaches have demonstrated efficacy in preclinical models. For example, a LOXi-ADC targeted to profibrotic macrophages, an anti–SPP1-AOC targeted to TREM2^+^ scar-associated macrophages, and an LNP siRNA anti–TGF-β receptor 1 targeted to hepatic stellate cells had preclinical efficacy in models of muscle, heart, and liver fibrosis, respectively (194–196). Multiomics datasets may provide additional means to identify robust cell-targeting arms. As RNA levels may not always correlate with protein expression, combining single-cell transcriptomics with proteomics is essential to validate cell surface protein expression (e.g., CITE-seq; ref. 142) and subcellular protein localization (197, 198) to yield selectivity.

### Depletion strategies of pathogenic players.

A promising concept is to “reset” the fibrotic niche by depleting established pathogenic cells, such as B cells, myofibroblasts, or profibrotic macrophages (199). Depletion strategies include antibody-dependent cell-mediated cytotoxicity, ADCs with cytotoxic payloads (typically microtubule or DNA topoisomerase inhibitors), T cell engagers (TCEs), or chimeric antigen receptors (CARs), which can be expressed in a variety of immune cells with clearance function (e.g., T, NK, and myeloid cells) (200, 201). While many of these strategies focus on treating tumors, the translation of B cell–depleting therapies such as B cell maturation antigen and CD19 TCEs and/or CAR T from oncology to inflammatory diseases is opening the door for such approaches in fibrotic diseases (202, 203). B cell depletion (e.g., rituximab) was as efficacious as the standard of care (i.e., cyclophosphamide) in diffuse systemic sclerosis, including in lung endpoints, to improve symptoms and lung function (204, 205). Preclinically, myofibroblasts’ depletion using ADCs or CAR T cells successfully achieved antifibrotic activity in hepatic and cardiac fibrosis, respectively (206, 207). Furthermore, there is growing evidence of epigenetic imprinting of injury in multiple cell types, including mesenchymal and myeloid cells. Cell depletion will remove the associated pathogenic functions and reduce epigenetic memory that contributes to accelerated progression following rechallenge (102, 208). Repopulation of cells educated in the homeostatic niche may support long-term remission.

## Conclusion

After a long pause following approval of the first two antifibrotic drugs, new waves of drugs covering a range of novel mechanisms are close to or exceeding clinical benefit expectations across a number of fibrotic diseases. This includes the first approvals for MASH, in which important therapeutic endpoints were finally achieved in some patients, leading to improvements in patients’ lives (14, 15), and the recent approval of nerandomilast (153) for IPF. The omics revolution is providing a deeper understanding of key mechanisms at the cellular and molecular level and revealing mechanistic hallmarks of fibrosis that span multiple tissues and fibrotic diseases. Omics readouts help clarify tolerability concerns with antifibrotic drugs by revealing essential overlapping homeostatic functions of individual targets. Omics data also reveal cell-selective targeting options and additional insights that drive new modality selection to improve the treatment of fibrotic diseases. Whether the enhanced biologic sensitivity afforded by omics and AI/ML can improve early detection of fibrogenesis, increase ability to detect therapeutic efficacy in trials, or open new windows into early intervention remains to be seen. Thus, the call to arms for omics technology applications will be to go beyond high-resolution phenotypic understanding of disease. Functional, kinetic, and dynamic insights on pathobiology will enable novel therapeutics and combinations to achieve rapid and robust resolution of fibrosis, amelioration of disease, and regeneration of homeostatic organ architecture and function.

## Conflict of interest

CL, KLD, KD, TAW, KMH, and TF are employees of Pfizer Inc.

## Version 1 07/08/2026

Electronic publication
